# Crystal Structure of BaCa(CO_3_)_2_ Alstonite Carbonate and Its Phase Stability upon Compression

**DOI:** 10.1021/acsearthspacechem.1c00032

**Published:** 2021-04-23

**Authors:** Raquel Chuliá-Jordán, David Santamaria-Perez, Javier Ruiz-Fuertes, Alberto Otero-de-la-Roza, Catalin Popescu

**Affiliations:** † Departamento de Física Aplicada-ICMUV, Universitat de València, MALTA Consolider Team, 46100 Valencia, Spain; ‡ DCITIMAC, Universidad de Cantabria, MALTA Consolider Team, 39005 Santander, Spain; § Departamento de Química Física y Analítica, Facultad de Química, Universidad de Oviedo, MALTA Consolider Team, 33006 Oviedo, Spain; ∥ CELLS-ALBA Synchrotron Light Facility, Cerdanyola del Vallès, 08290 Barcelona, Spain

**Keywords:** alstonite, BaCa(CO_3_)_2_, crystal structure, carbonate, phase transition, high pressure, synchrotron X-ray diffraction, DFT calculations

## Abstract

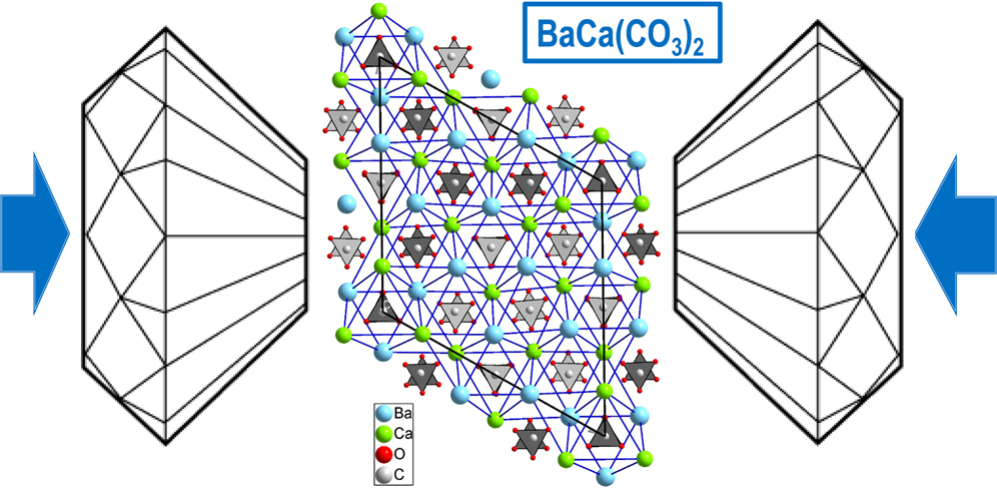

New single-crystal X-ray diffraction experiments and density functional theory (DFT) calculations reveal that the crystal chemistry of the CaO–BaO–CO_2_ system is more complex than previously thought. We characterized the BaCa(CO_3_)_2_ alstonite structure at ambient conditions, which differs from the recently reported crystal structure of this mineral in the stacking of the carbonate groups. This structural change entails the existence of different cation coordination environments. The structural behavior of alstonite at high pressures was studied using synchrotron powder X-ray diffraction data and ab initio calculations up to 19 and 50 GPa, respectively. According to the experiments, above 9 GPa, the alstonite structure distorts into a monoclinic *C*2 phase derived from the initial trigonal structure. This is consistent with the appearance of imaginary frequencies and geometry relaxation in DFT calculations. Moreover, calculations predict a second phase transition at 24 GPa, which would cause the increase in the coordination number of Ba atoms from 10 to 11 and 12. We determined the equation of state of alstonite (*V*_0_ = 1608(2) Å^3^, *B*_0_ = 60(3) GPa, *B*′_0_ = 4.4(8) from experimental data) and analyzed the evolution of the polyhedral units under compression. The crystal chemistry of alstonite was compared to that of other carbonates and the relative stability of all known BaCa(CO_3_)_2_ polymorphs was investigated.

## Introduction

Abundance of carbon on the earth’s surface significantly differs from the solar pattern.^1^ The deficiency of carbon on the earth’s surface can be explained by an efficient deep carbon ingassing during the last billion years of earth’s history.^2^ That is, missing carbon would be stored in our planet’s interior in stable reduced or oxidized carbon forms. The most probable candidates for the oxidized carbon species in the mantle are carbonates, which enter via subduction processes at convergent boundaries.

Magnesium and calcium carbonates are the most abundant on the earth’s surface. Ca-containing CaCO_3_ calcite and CaMg(CO_3_)_2_ dolomite are the principal carbonate minerals in sedimentary rocks.^3^ MgCO_3_ magnesite, on the other hand, has been suggested as the main host of oxidized carbon throughout the mantle due to its structural stability.^4^ In fact, the stability of both Ca and Mg simple carbonate minerals differs greatly. While magnesite is stable throughout a large pressure and temperature range (only a dense polymorph has been discovered above 115 GPa and 2100 K),^4,5^ calcite undergoes several phase transitions under compression.^6,7^ In the case of dolomite, two high-pressure (HP) polymorphs have been reported.^8^ Despite the fact that these carbonates have been extensively studied, the crystal chemistry of calcium carbonates and, in particular, the local environment around the Ca atoms needs to be well understood. For instance, pure CaCO_3_ has been naturally found at ambient conditions in three structural forms: (i) calcite, the rhombohedral stable phase, where the Ca atoms are octahedrally coordinated by O atoms, (ii) aragonite, an orthorhombic phase, where the Ca atoms have nine O neighbor atoms, and (iii) vaterite, a rare phase with partial atomic occupation factors, where the Ca atoms are 8-fold coordinated in average. The explanation of such behavior has often been oversimplified in the literature to a mere question of the Ca^2+^ cation size, which would delimit the border of the calcite- to aragonite-type structures.

The presence of other cations like alkaline (Na^+^, K^+^), alkaline-earth (Mg^2+^, Sr^2+^, Ba^2+^), or transition metals (Mn^2+^, Zn^2+^, Fe^2+^) in the carbonate structure, as well as the existence of additional anions that could provide negative charge, conditions the Ca local environments. Thus, whereas CaM(CO_3_)_2_ (M = Mg, Mn, Zn, Fe) present Ca-centered octahedra,^8−11^ the different Ca–Mg stoichiometry of huntite CaMg_3_(CO_3_)_4_ entails the formation of Ca-centered trigonal prisms.^12^ In calcium carbonates that include Na^+^, K^+^, Sr^2+^, Ba^2+^ cations, Ca coordination varies from 6 to 9 depending on the structure or even within the same structure.^13−22^ Naturally occurring calcium silicate-carbonates, for instance, present a large variety of Ca atom environments, ranging from 6-fold to 9-fold coordination.^23,24^ Note that the different classes of irregular cation-centered oxygen polyhedra exhibit a wide range of volumes and bulk moduli, which suggests that other divalent cation species could be accommodated in these sites. Therefore, the study of the atomic arrangements in different Ca carbonate systems and their behavior at high pressures could provide insight into the nature of the Ca–(CO_3_) interactions and potential chemical substitution at inner earth conditions.

The double carbonate CaBa(CO_3_)_2_ exists naturally as three different polymorphs: monoclinic barytocalcite,^19^ and trigonal intimately related alstonite^20^ and paralstonite^21^ phases. A new monoclinic polymorph has also been synthesized.^22^ As it occurs in CaCO_3_, Ca atoms in CaBa(CO_3_)_2_ adopt 6-fold or 8-fold coordination depending on the polymorph. This work determines an ambient condition alstonite structure that differs from a previously reported solution^20^ and reports its experimental high-pressure behavior. Density functional theory (DFT) calculations of the different CaBa(CO_3_)_2_ phases shed light on their relative thermodynamical stabilities upon compression. The evolution of the lattice parameters and atomic coordinates at high pressure shows the change of cation environments and allows determining both polyhedra and bulk compressibilities.

## Experimental Details

Naturally occurring alstonite samples from the Fallowfield mine, in Northumberland (U.K.), were kindly provided by the Yale Peabody Museum (Specimen YPM MIN 034129). A few crystals were optically selected under the microscope. Some of them were crushed to obtain a fine white powder. Qualitative chemical analyses were done on a Philips XL30 scanning electron microscope using energy-dispersive X-ray spectroscopy. According to them, the chemical composition of our alstonite sample was Ba_0.96(3)_Sr_0.05(1)_Ca_0.99(6)_(CO_3_)_2_. We solved the alstonite structure from angle-dispersive single-crystal X-ray diffraction (XRD) data collected on a Bruker D8 Venture diffractometer at ambient conditions using Mo Kα radiation. Indexing, data reduction, and empirical absorption correction were performed using APEX3 software. Structure solutions and structural refinements were performed with SHELXT^25^ and SHELXL,^26^ respectively, operated using the WinGX interface.^27^ We found that the sample has a structure slightly different than the one recently reported by Bindi et al.^20^ The structural solution will be briefly described later.

High-pressure angle-dispersive powder XRD experiments were conducted at the MSPD beamline of the ALBA-CELLS Synchrotron Light Source^28^ using a monochromatic incident beam of 0.4246 Å. HP measurements were performed using a diamond-anvil cell (DAC), a technique that allows to strongly modify and subsequently analyze the atomic interaction in solids.^29,30^ The alstonite sample was placed in a stainless-steel gasket cavity inside the membrane DAC along with the Cu powder for pressure determination^31^ and a 4:1 mixture of methanol–ethanol was used as a pressure-transmitting medium.^32^ Diffraction patterns were collected at different pressures for 20 s up to 19 GPa. The LaB_6_ powder was used for distortion correction, and integration to conventional 2θ-intensity data was carried out with Dioptas software.^33^ The indexing and refinement of the powder patterns were performed using the Unitcell^34^ and Powdercell^35^ program packages.

## Computational Details

Calculations were carried out using the projector augmented wave (PAW) method^36^ implemented in Quantum ESPRESSO.^37^ The number of valence electrons in the atomic data sets are: 10 (Ba), 10 (Ca), 4 (C), and 6 (O). We used a 100 Ry plane-wave cutoff for the Kohn–Sham states and a 1000 Ry cutoff for the electron density. The functional used was B86bPBE,^38,39^ combined with the exchange–hole dipole moment (XDM) model for dispersion.^40,41^ To confirm the results, we also used the PBEsol functional,^42^ which gives equivalent results to B86bPBE-XDM. We explored the convergence of the total energy and stress tensor with respect to *k*-point grid size. Satisfactory convergence was achieved using the following (shifted) *k*-point grids: 3 × 3 × 3 (barytocalcite), 3 × 3 × 3 (paralstonite), 1 × 1 × 4 (alstonite), 1 × 1 × 4 (*C*2 HP-alstonite), and 3 × 3 × 3 (*C*2 synthetic phase, ref (22).).

Geometry optimizations were carried out with tight convergence parameters (10^–5^ Ry in the energies and 10^–4^ Ry/bohr in the forces). For each phase, the equilibrium geometry was determined at zero pressure and at 50 GPa, and a uniform volume grid was built between the two structures containing 41 points. Constant-volume geometry optimizations were run at each point in the volume grid, and the resulting energy–volume equation of state was input into the gibbs2 program^43,44^ to fit analytical equations of state and determine the phase stability under pressure. We also calculated the Gamma for alstonite using the phonopy software.^45^

## Results

### Crystal Structure of Alstonite at Ambient Conditions

**Figure 1:**
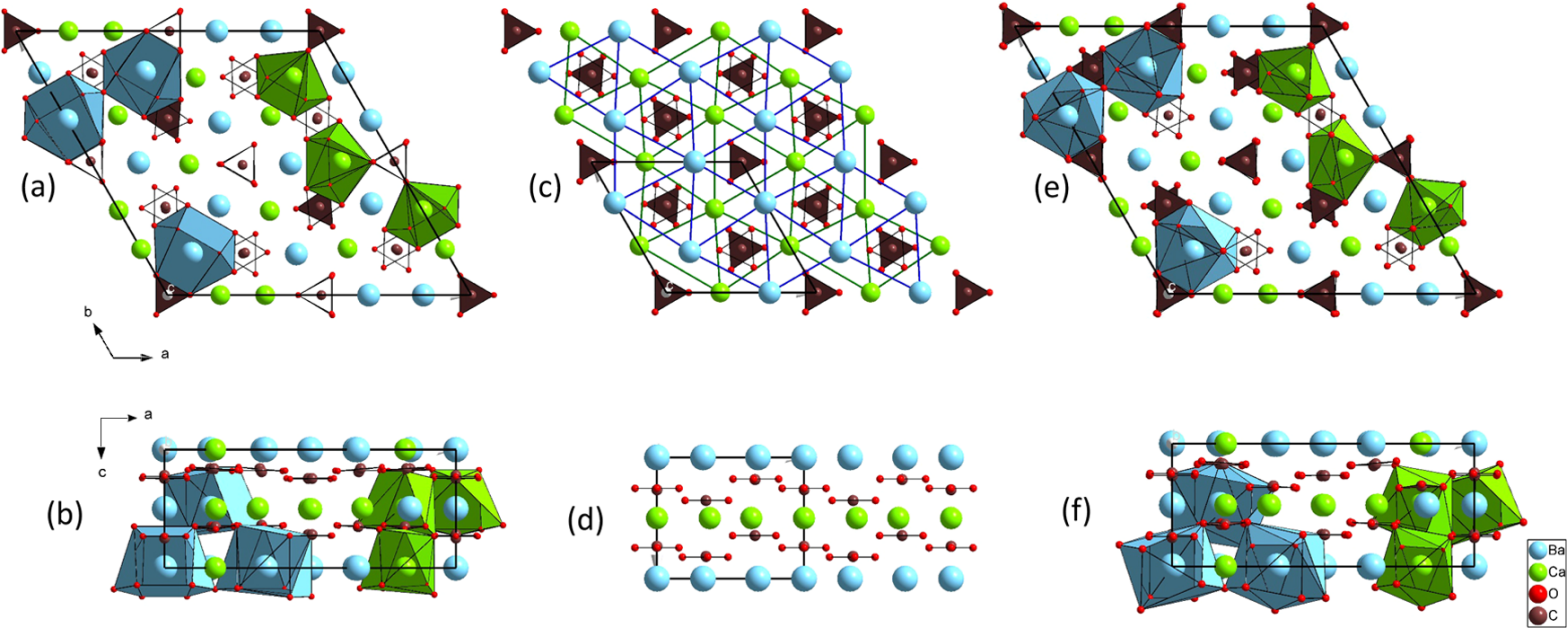
Projections along the *c* and *b* axes of the crystalline BaCa(CO_3_)_2_ structures of (a, b) trigonal *P*31*m* alstonite reported by Bindi et al.,^20^ (c, d) trigonal *P*321 paralstonite,^21^ and (e, f) trigonal *P*321 alstonite [this work]. Thick black lines indicate the unit-cell. Cyan, green, brown, and small red spheres represent Ba, Ca, C, and O atoms, respectively. In alstonite representations (a, b, e, and f), the three types of [BaO_10_] and [CaO_8_] polyhedra are depicted to illustrate the differences in shape caused by the location of the [CO_3_] groups in both structural solutions. In paralstonite representation (c, d), the Ba–Ba and Ca–Ca distances are shown to illustrate the hexagonal metallic layers existing in carbonate compounds.

Since its first identification in the mid-XIX century,^46^ the crystal structure of BaCa(CO_3_)_2_ has remained unknown until very recently, when Bindi et al. reported that it could be described by a trigonal *P*31*m* space group (SG) with lattice parameters *a* = 17.4360(6) Å and *c* = 6.1295(2) Å (*V* = 1613.80(9) Å^3^, *Z* = 12).^20^ The crystal structure of reported alstonite is depicted in Figure 1a,b. It consists of two different hexagonal layers of cations perpendicular to the *c* axis (at *z*/*c* = 0 and 0.5) stacked in a ...ABAB... conformation, one is Ba-rich with a ratio Ba/Ca = 3 in cation sites, and in the other layer the ratio Ba/Ca is the inverse. As it can be seen in the *ac* projection, the carbonate groups are located among the cation layers and are arranged parallel to these, the [CO_3_] units being at two different heights between the Ba/Ca layers (at *z*/*c* ∼ 1/4, 1/3 or 3/4, 5/6). The chemical composition of the cation layers in alstonite is one of the main differences with respect to the paralstonite polymorph,^21^ depicted in Figure 1c,d. The latter is characterized by alternating layers of Ca and Ba atoms along the *c* axis, which shows that the structure could be described by a half-length *a* axis. The other difference is the arrangement of the [CO_3_] groups, in which parastonite are staggered at three heights between cation layers (at *z/c* ∼ 1/6, 1/4, 1/3 or 2/3, 3/4, 5/6). Both crystal structures have the Ba atoms in 10-fold coordination and Ca atoms in 8-fold coordination, but the shape of the cation-centered O polyhedra are different. For instance, the difference between shorter and longer Ca(1)–O distances in the [Ca(1)O_8_] polyhedron of the reported *P*31*m* alstonite (2.184(13) – 2.752(7) Å)^20^ is significantly larger than that observed in paralstonite [CaO_8_] polyhedra (2.36(2)–2.59(1) Å).^21^

As a previous step to the study of our alstonite sample, we selected a clean, transparent, and crack-free crystal with dimensions 100 × 100 × 20 μm^3^ under the optical microscope to perform an ambient condition single-crystal XRD experiment and solve its structure. After the data collection, the indexation confirmed a hexagonal symmetry with lattice parameters *a* = 17.460(3) Å and *c* = 6.125(2) Å. The data integration suggested noncentrosymmetric space groups *P*6̅2*m* (*R*_int_ = 0.1445), *P*31*m* (*R*_int_ = 0.1385), and *P*321 (*R*_int_ = 0.1026). Considering that the structure had been recently solved by Bindi et al., in space group *P*31*m*, we solved the structure of our alstonite sample in *P*31*m* and *P*321 with the lowest internal *R* value. For the structural solution, we employed SHELXT, which located all of the metallic ions and a large number of oxygens and carbons, yielding in both space groups *R*1 values of 0.158 and 0.143 for *P*31*m* and *P*321, respectively. Further refinement cycles with the SHELXL program helped to locate the remaining atoms in both space groups obtaining, with the isotropic refinement of all atoms, R1 values of 0.0732 (*P*321) and 0.0908 (*P*31*m*). Even though these ordered solutions with full-site occupations seemed reasonable, both refinements showed residual charge densities for most positions and, in particular, in one of the Ca positions. We refined then allowing site occupancy for this Ca position with some Sr (also present in our sample) considerably lowering the *R*1 value but providing unrealistic atomic distances in both space groups. Hence, we stepped back and continued with fully ordered refinement but this time refining the metallic atoms anisotropically. This yielded residual values of *R*1 = 0.0643 for 1740 diffraction peaks whose Fo > 4σ(Fo) and 106 refined parameters in *P*321, and *R*1 = 0.0837 for 1816 diffraction peaks whose Fo > 4σ(Fo) and 117 refined parameters in *P*31*m*. Crystal refinement details of both space groups are shown in Table 1. Hence, we conclude that the structure of our alstonite sample is better described in space group *P*321 with the residual charge density being most probably due to uncounted occupational disorder.

**Table 1:** Parameters and Results of Single-Crystal XRD Data Collection, Data Reduction, and Crystal Refinement

	**Crystal Data**	
chemical formula	BaCa(CO_3_)_2_	BaCa(CO_3_)_2_
cell parameters		
*a*(Å)	17.459(3)	17.459(3)
*c*(Å)	6.125(2)	6.125(2)
*V*(Å^3^)	1616.9(8)	1616.9(8)
*Z*	12	12
space group	*P*321	*P*31*m*
ρ(g/cm^3^)	3.668	3.668

	**Data Collection**	
temperature (K)	299(2)	299(2)
λ(Å)	0.71073	0.71073
reflection range		
	–22 ≤ *h* ≤ 22	–22 ≤ *h* ≤ 22
	–20 ≤ *k* ≤ 21	–20 ≤ *k* ≤ 21
	–7 ≤ *l* ≤ 7	–7 ≤ *l* ≤ 7
no. of reflections obs	12 000	12 000
unique reflections obs	2411	2532
rint, obs	0.1026	0.1385

	**Refinement**	
no. of parameters	106	117
*R*1	0.0643	0.0837
wR2	0.2148	0.2713
GooF	1.036	1.006

The new *P*321 structure of alstonite is shown in Figure 1e,f. It is intimately related to paralstonite. In paralstonite, the metal atoms are arranged alternating pure Ca and pure Ba hexagonal layers and the unit-cell parameters are *a* = 8.692(3) and *c* = 6.148(4) Å (*Z* = 3).^21^ In the alstonite structure, the Ba-rich layer contains 3/4 parts of Ba atoms and 1/4 of Ca atoms, while in the Ca-rich layer is the opposite. Thus, the Ca(2) atoms at 3e sites and Ba(3) atoms at 3f sites occupy positions in the Ba- and Ca-rich layers, respectively. The resulting cation layers are identical to those reported by Bindi et al. using the SG *P*31*m* to describe the structure. Between layers, the carbonate groups lie approximately parallel to the *ab* plane in three different levels, as opposed to the two levels reported using the *P*31*m* structural model. The cation rearrangement with respect to paralstonite entails that certain carbonate groups appear rotated as a consequence of the accommodation of cations of different sizes in adjacent layers. For instance, half of the [CO_3_] groups at *y*/*b* = 0 and all of the [CO_3_] groups at *y*/*b* = 1/2 are rotated 60° relative to those of paralstonite, in such a way that all Ba and Ca atoms could adopt the 10-fold and 8-fold coordination, respectively. This structural arrangement can only be described with a unit-cell where the *a* axis is doubled with respect to parastonite (*Z* = 12). Note that alstonite defined within the *P*321 SG provides more uniform Ca–O distances (3.31(1)–2.65(1) Å) than the *P*31*m* structural model (see above). The atomic coordinates of the refined structure are collected in Table 2.

**Figure 2:**
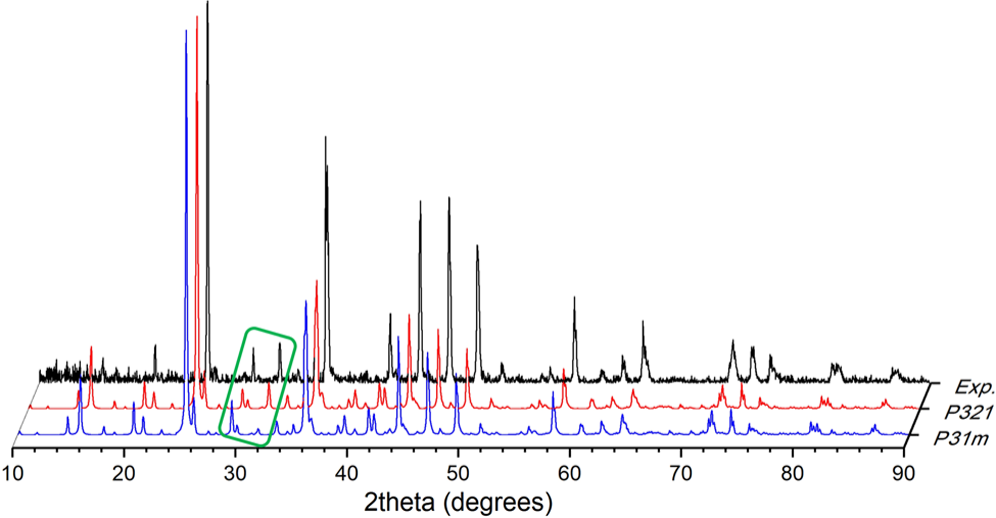
Experimental XRD pattern of alstonite (black) and calculated profiles of both *P*31*m* (blue)[20] and *P*321 (red) [this work] trigonal phases reported for alstonite. The patterns correspond to Cu Kα wavelength (λ_Kα1_ = 1.540598 and λ_Kα2_ = 1.544426 Å). Both structural models give rise to similar diffraction intensities, except in the 2θ range 29–32° (region within the green rectangle). A slightly better profile match is obtained with the *P*321 structure.

The correct determination of the crystal structure of our alstonite mineral is supported by the slightly better match of the experimental powder XRD pattern with the *P*321 model (see Figure 2). The diffraction peak intensities calculated with both the *P*321 and the *P*31*m* structural models are similar in the whole 2θ range, except between 29 and 32° (Cu Kα wavelength). The reflections (202) and (022) modeled by the *P*321 structural description explain the experimental diffraction peak at 31.6° better.

**Figure 3:**
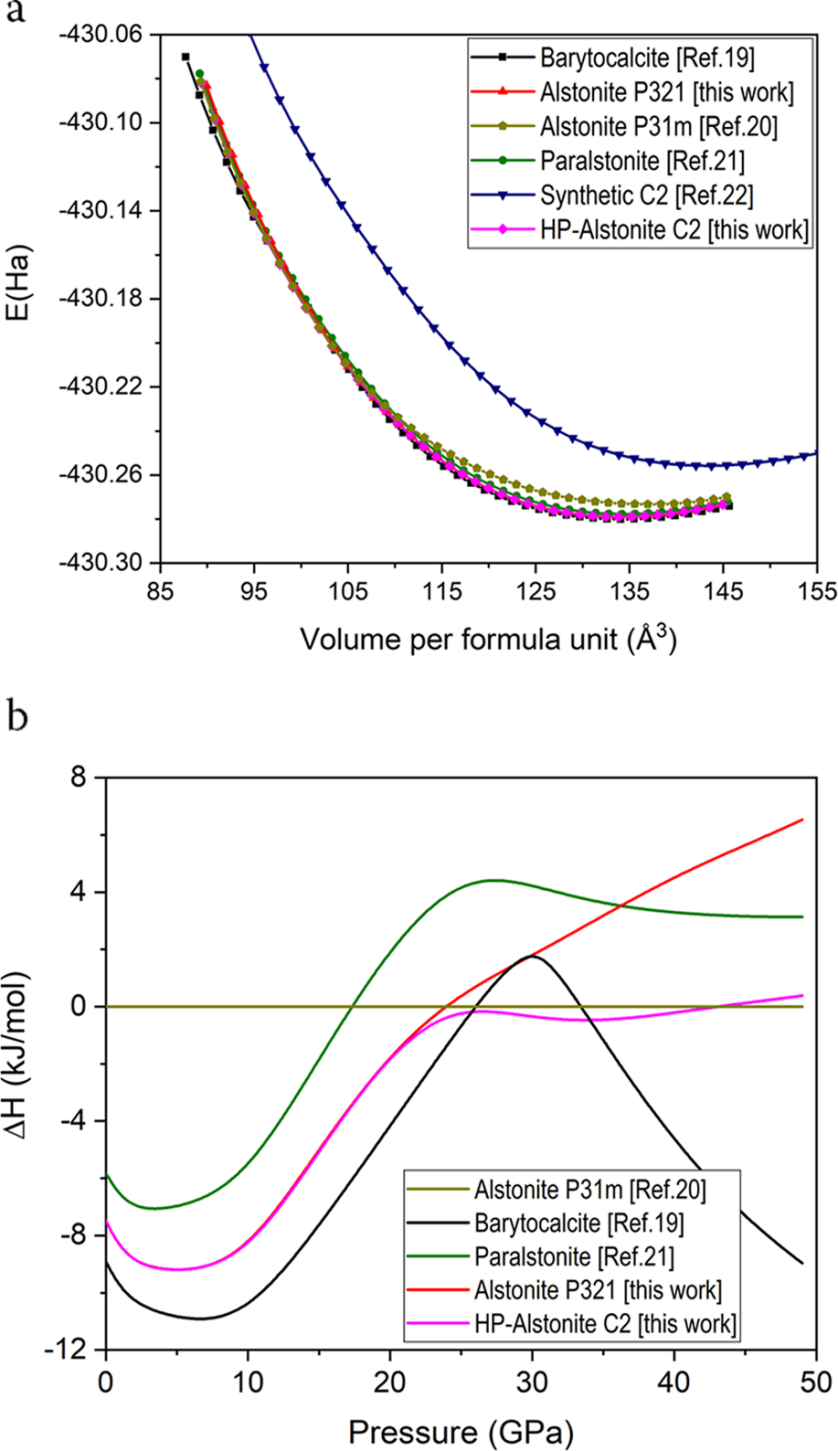
(a) Cohesive energy as a function of the volume per BaCa(CO_3_)_2_ formula unit for the *P*2_1_/*m* barytocalcite,^19^*P*32 paralstonite,^21^ synthetic *C*2,^22^*P*31*m* alstonite,^20^ and *P*321 alstonite [this work]. (b) Enthalpy difference as a function of pressure showing the higher stability of *P*321 alstonite with respect to *P*31*m* alstonite. The *P*31*m* alstonite^20^ phase has been taken as a reference.

Additionally, we carried out DFT calculations of all of the known BaCa(CO_3_)_2_ polymorphs up to 50 GPa, that is, *P*2_1_/*m* barytocalcite,^19^*P*321 parasltonite,^21^ the synthetic *C*2,^22^ and the *P*31*m*^20^ and *P*321 [this study] alstonite phases. Figure 3a,b shows the energy as a function of calculated volume curves and the enthalpies calculated for each phase referred to the enthalpy of alstonite *P*31*m* phase,^20^ respectively. The enthalpies of alstonite *P*321, paralstonite, and barytocalcite are very similar over the studied pressure range, within 3 KJ/mol (0.08 eV) per formula unit of each other. This indicates that either of these phases may be observable under the proper experimental conditions, and it is not possible to predict on the basis of stability calculations alone whether one or the other will appear. At equilibrium and zero pressure, the *P*31*m* alstonite variant^20^ has higher energy than the three aforementioned phases (approx. 9 KJ/mol, or 0.19 eV per formula unit higher than barytocalcite), which indicates that this phase is less stable. The synthetic *C*2 phase is unstable at all pressures relative to any of the other phases. This discussion on the stability of the different BaCa(CO_3_)_2_ polymorphs is based on enthalpy differences alone, the entropic effects being disregarded (they may be important at high temperatures).

### Structural Properties of Alstonite under Pressure

Quasi-hydrostatic compression increases density and alters the interatomic interactions, which may lead to phase transitions to minimize the overall free energy of the system.^47^ In this section, we study the structural response of alstonite to increasing pressure.

**Figure 4:**
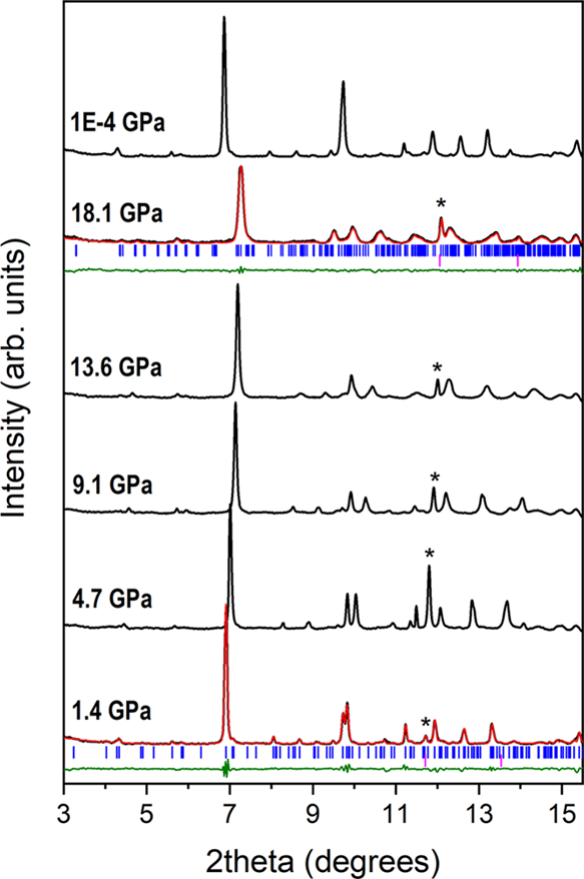
Powder XRD patterns of BaCa(CO_3_)_2_ alstonite at different pressures. The pattern of the recovered alstonite sample is shown on top. Asterisks denote the diffraction maxima of copper, the internal pressure gauge.

High-pressure synchrotron powder XRD patterns present texturing effects due to uneven crystal sizes. This effect causes inaccuracies in the relative intensities of the diffraction maxima and precludes full structural refinements. Only LeBail refinements could be performed. Under compression, the diffraction peaks shift to higher angles as expected for a decrease of interplanar distances, but no additional maxima are observed (see Figure 4). XRD peaks of our patterns were indexed with the trigonal unit-cell of alstonite in all of the pressure range of this study, which suggests that alstonite is stable up to 19 GPa. The indexed lattice parameters and unit-cell volumes at different pressures are collected and shown in Table 1S of the Supporting Material.

**Figure 5:**
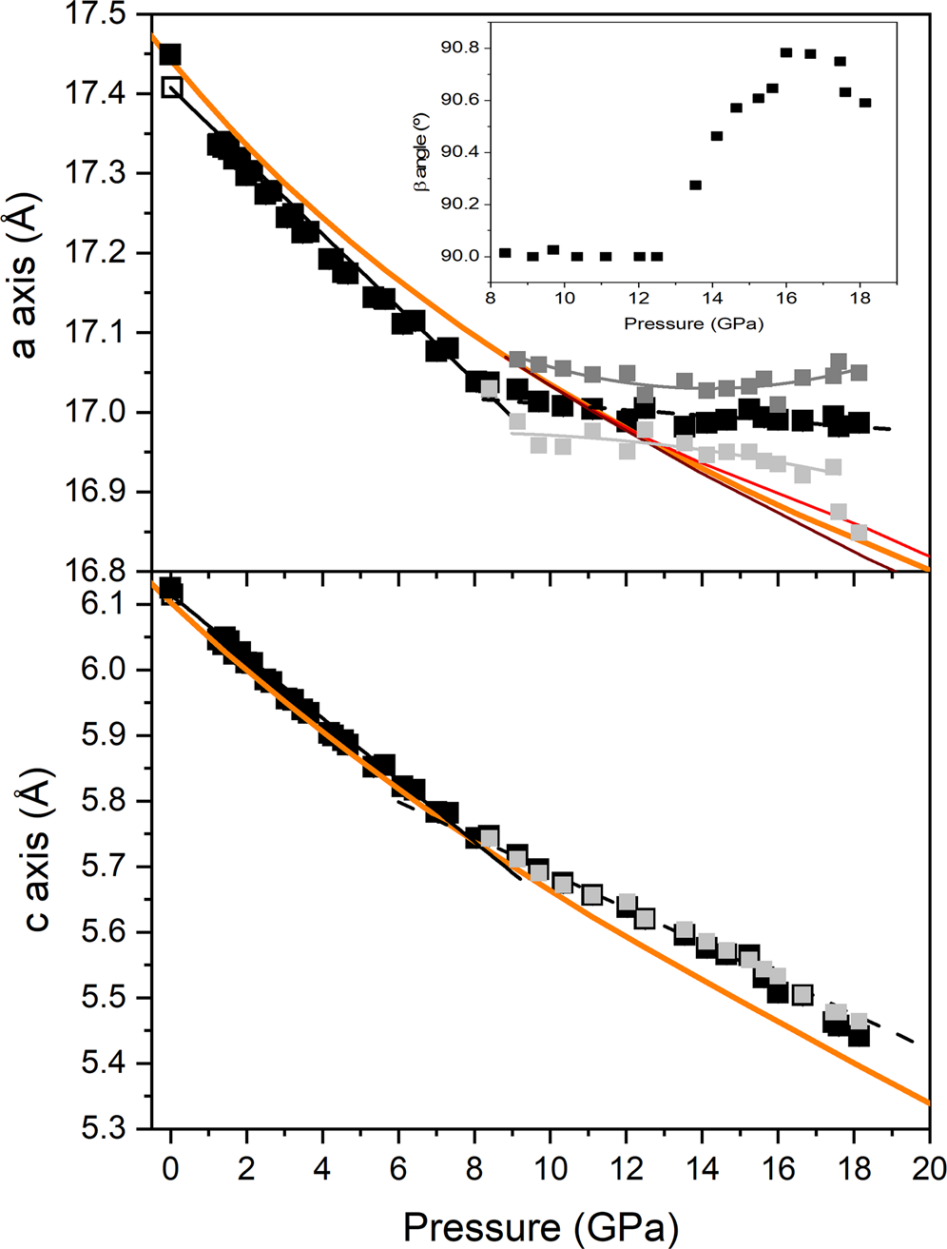
Pressure dependence of the *a* (top) and *c* (bottom) lattice parameters of BaCa(CO_3_)_2_ alstonite. Upstroke experimental data are depicted as solid squares, whereas the data of the recovered sample are depicted with empty squares. Fits to experimental *P*321 data in the pressure ranges 0–9 and 9–19 GPa are represented as solid and dashed black lines, respectively. Lattice parameters from monoclinic *C*2 indexations are depicted in dark and light gray symbols. DFT data are represented as solid orange lines.

**Figure 6:**
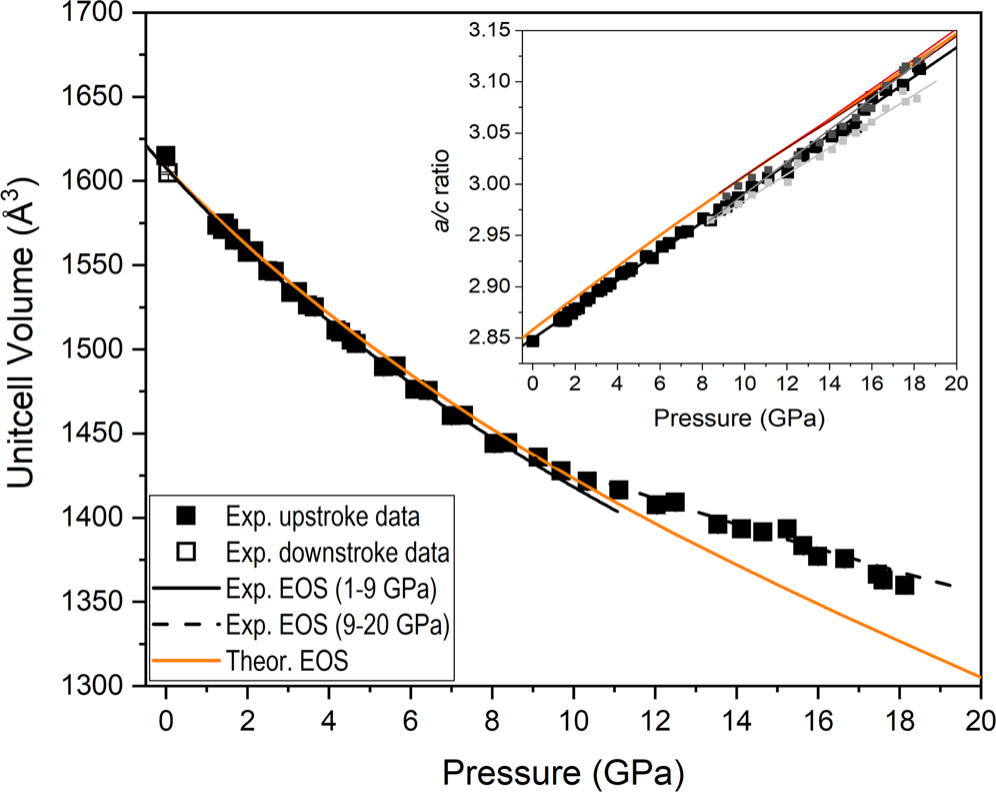
Pressure dependence of the unit-cell volume of BaCa(CO_3_)_2_ alstonite. Upstroke experimental data are depicted as solid squares, whereas the data point corresponding to the recovered sample is depicted with an empty square. Fits to experimental data in the pressure ranges 0–9 and 9–19 GPa (*P*321 and *C*2 unit-cells yield similar volumes per formula unit) are represented as solid and dashed black lines, respectively. DFT data are represented as solid orange lines. Inset: Evolution of the *a*/*c* axes ratio as a function of pressure.

The evolution of the unit-cell parameters (Figure 5) and volume (Figure 6) as a function of pressure presents a good overall agreement with our ab initio total-energy calculations below 9 GPa. In the following, theoretical values are denoted in parentheses. The lattice parameters of the trigonal *P*321 unit-cell vary smoothly with increasing pressure up to 9 GPa, which supports the absence of phase transitions in this pressure range. The absolute experimental contractions for the *a*- and the *c*-axes at this pressure are 0.438(7) and 0.403(5) Å, respectively. Experimental (theoretical) axial linear compressibilities for alstonite in the 0–9 GPa range are β_a_ = 2.51(5) × 10^–3^ (2.48(7) × 10^–3^) and β_c_ = 7.20(12) × 10^–3^ (7.47(13) × 10^–3^) GPa^–1^ and indicate strong axial anisotropy. The axial compression ratio defined as β_c_/β_a_ is 2.87(7) (3.01(8)). This result shows that the *c* axis is approximately 3 times more compressible than the *a* axis. The *a*/*c* axes ratio increases with pressure according to the expression *a*/*c* = 2.850(1) – 0.0143(2)·P (see the inset of Figure 6). This response to external pressure arises from the fact that the relatively incompressible [CO_3_] carbonate units are arranged approximately parallel to the *ab* plane, whereas the compressibility of the *c* axis can be directly attributable to the [BaO_10_] and [CaO_8_] polyhedral compression. A third-order Birch–Murnaghan equation of state (BM-EoS) fit^48^ to our P–V data set yields a zero-pressure volume *V*_0_ = 1608(2) Å^3^, a bulk modulus of *B*_0_ = 60(3) GPa, and a bulk modulus first-pressure derivative of *B*_0_′ = 4.4(8). These experimental results compare well with those obtained from theoretical calculations: *V*_0_ = 1608.0(1) Å^3^, *B*_0_ = 64.25(4) GPa, and *B*_0_′ = 3.99(1). The bulk modulus lies in between those of the two end-member carbonates: 67(2) GPa for CaCO_3_ calcite,^49^ 66.5(7) GPa (*B*′_0_ = 5.0(1)) for CaCO_3_ aragonite^50^ and 48(1) GPa for BaCO_3_ witherite,^51^ and it is comparable to the 62.7(6) GPa of SrCO_3_ strontianite.^51,52^ In other words, our data evidences that BaCa(CO_3_)_2_ alstonite is more compressible than all of the divalent metal carbonates and silicate-carbonates except witherite.^12,48,52−54^

**Figure 7:**
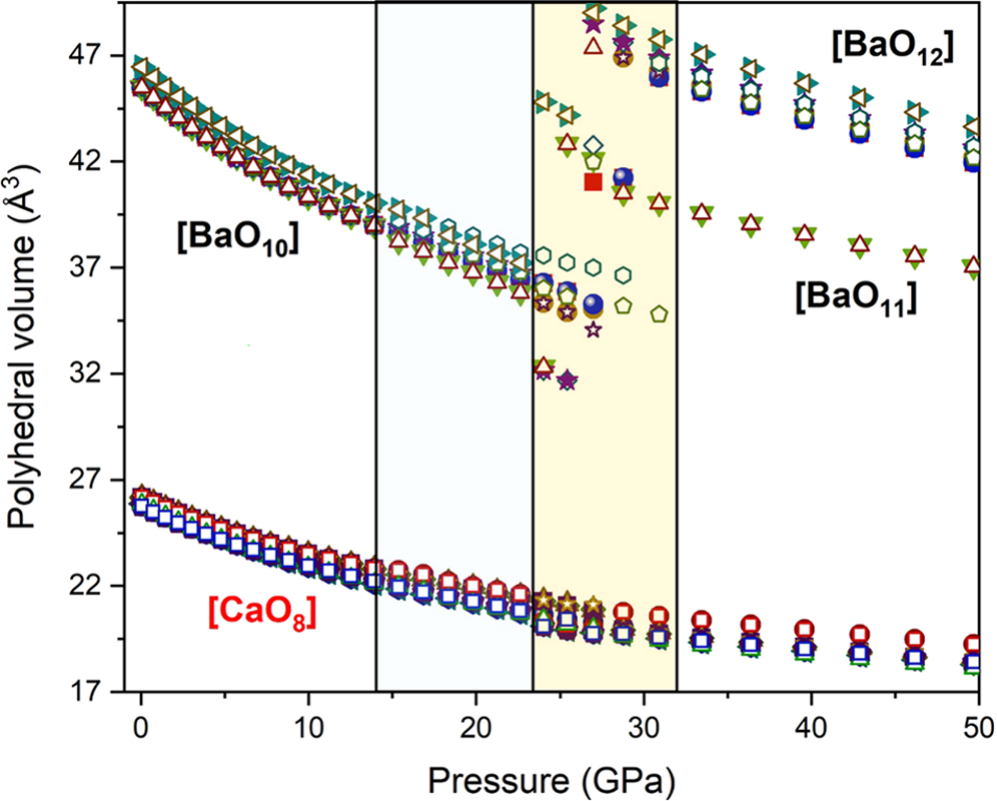
Representation of the evolution under compression of the different polyhedral unit volumes of BaCa(CO_3_)_2_ alstonite according to DFT calculations. Above 24 GPa, the Ba atoms increase their coordination numbers from 10 to 11 (17%) and 12 (83%).

Taking into account the good agreement found between experimental and theoretical data in (i) lattice parameters and atomic positions at ambient conditions (see Tables 2 and 3) and (ii) the unit-cell compressibility behavior within the 0–9 GPa pressure range, we use data from our DFT simulations to study the variation of bond distances and polyhedral compressibilities with pressure. The Ba- and Ca-centered polyhedral volumes of BaCa(CO_3_)_2_ alstonite vary smoothly with pressure in that range (see Figure 7) and give the bulk moduli shown in Table 4. It can be seen that the bulk moduli of both the Ba-centered and Ca-centered O polyhedra are similar (ranging from 60.5 to 68.2 GPa), but the *B*′_0_ values are higher for [CaO_8_]. This means that these polyhedra become progressively more incompressible with increasing pressure.

**Table 2:** Atomic Coordinates and Thermal Displacements (*U*_eq_/*U*_iso_) of *P*321 BaCa(CO_3_)_2_ Alstonite from Single-Crystal XRD Data at Ambient Conditions

**atom**	***x***	***y***	***z***	***U*_eq_**	***U*_iso_**
Ba01	0.15617 (16)	0.50234(15)	0.9997(3)	0.0111(5)	
Ba02	0.3385(16)	0.33825(16)	0	0.0082(7)	
Ba03	0.15772(17)	0.15772(17)	0.5	0.0117(7)	
Ca01	0.1798(4)	0.6818(4)	0.4999(9)	0.0022(12)	
Ca02	0	0.1777(7)	0	0.017(3)	
Ca03	0	0.328(7)	0.5	0.012(2)	
O01	0.0715(12)	0.9966(12)	0.756(3)		0.013(4)
O02	0.2324(12)	0.4055(12)	0.177(3)		0.008(4)
O03	0.1617(13)	0.4105(12)	0.640(3)		0.009(4)
O04	0.9950(12)	0.4278(13)	0.740(3)		0.016(4)
O05	0.7379(15)	0.4102(14)	0.175(3)		0.017(5)
O06	0.0843(13)	0.3268(13)	0.168(3)		0.013(4)
O07	0.2359(14)	0.3393(13)	0.651(3)		0.012(4)
O08	0.9253(12)	0.5081(12)	0.743(3)		0.019(4)
O09	0.0748(13)	0.5755(13)	0.742(3)		0.023(4)
O10	0.1593(13)	0.2559(14)	0.139(3)		0.012(5)
O11	0.0883(13)	0.2642(13)	0.667(3)		0.012(5)
O12	0.6594(16)	0.4027(15)	0.646(4)		0.025(6)
C01	0.66667	0.33333	0.177(7)		0.002(9)
C02	0.9996(11)	0.5054(11)	0.740(3)		0.001(5)
C03	0.161(2)	0.3360(18)	0.657(4)		0.008(5)
C04	0.66665	0.33332	0.652(8)		0.009(11)
C05	0	0.3283(19)	0.165(4)		0.013(6)
C06	0	0	0.762(7)		0.022(19)

**Table 3:** Atomic Coordinates of *P*321 BaCa(CO_3_)_2_ Alstonite at Ambient Pressure from DFT Calculations (*a* = 17.4419 Å and *c* = 6.1030 Å)

**atom**	***x***	***y***	***z***
Ba01	0.1552	0.5028	0.9967
Ba02	0.3370	0.3370	0.0000
Ba03	0.1584	0.1584	0.5000
Ca01	0.1821	0.6842	0.4965
Ca02	0.0000	0.1794	0.0001
Ca03	0.0000	0.3264	0.5001
O01	0.0714	0.9946	0.7561
O02	0.2310	0.4047	0.1745
O03	0.1603	0.4117	0.6288
O04	0.9958	0.4298	0.7412
O05	0.7372	0.4110	0.1826
O06	0.0820	0.3268	0.1672
O07	0.2372	0.3404	0.6441
O08	0.9264	0.5085	0.7460
O09	0.0746	0.5780	0.7377
O10	0.1587	0.2560	0.1399
O11	0.0880	0.2642	0.6668
O12	0.6614	0.4049	0.6451
C01	0.6667	0.3333	0.1857
C02	0.9990	0.5055	0.7349
C03	0.1621	0.3383	0.6504
C04	0.6667	0.3333	0.6491
C05	0.1580	0.3297	0.1639
C06	0.0000	0.0000	0.7628

**Table 4:** Bulk and [BaO_10_] and [CaO_8_] Polyhedral Compressibilities in *P*321 BaCa(CO_3_)_2_ Alstonite

	***V***_**0**_**(Å**^**3**^**)**	***B***_**0**_**(GPa)**	***B*′**_**0**_
BaCa(CO_3_)_2_ experiment	1608(2)	60(3)	4.4(8)
BaCa(CO_3_)_2_ theory	1608.0(1)	64.25(4)	3.99(1)
[Ba(1)O_10_]	45.531(6)	63.8(3)	4.37(5)
[Ba(2)O_10_]	45.579(4)	65.3(2)	4.31(3)
[Ba(3)O_10_]	46.501(3)	66.4(2)	4.43(3)
[Ca(1)O_8_]	26.208(5)	68.2(4)	5.50(8)
[Ca(2)O_8_]	25.899(5)	60.5(3)	4.71(6)
[Ca(3)O_8_]	25.773(4)	64.5(3)	5.00(6)

Above 9 GPa, the diffraction peaks of the XRD patterns could also be roughly explained with the trigonal unit-cell of alstonite. The *a* lattice parameter would become almost incompressible (see Figures 5 and 6), whereas the O–O contacts between two neighboring [CO_3_] units parallel to the *ab* plane increase their stiffness. As it occurs in other common layered materials, it is most compressible along the stacking axis than along a perpendicular direction. No volume discontinuities were observed in the studied pressure range. It is noticeable however that the width of several diffraction peaks, particularly those at higher 2θ angles, increases significantly above this pressure in the XRD pattern profiles. This fact could be due to the presence of nonhydrostatic stresses that could lead to the formation of a lower symmetry phase via a second-order symmetry-reduction phase transition, consequence of a lattice dynamical instability.

Phonon frequencies were calculated using the frozen-phonon method (implemented in Phonopy^45^) for the *P*321 alstonite phase at nine points between zero and 20 GPa. The appearance of imaginary phonon frequencies indicates that a dynamical instability develops in this phase at around 15 GPa. The eigenvector corresponding to one of the imaginary-frequency modes at 20 GPa was used to perturb the *P*321 structure and a subsequent fixed-volume geometry relaxation was carried out, which resulted in a broken-symmetry *C*2 phase with lower enthalpy than the *P*321 phase at high pressure. The *C*2 phase converges to the same structure as *P*321 alstonite at low pressure, while it diverges from it in the pressure range between 15 and 20 GPa.

**Figure 8:**
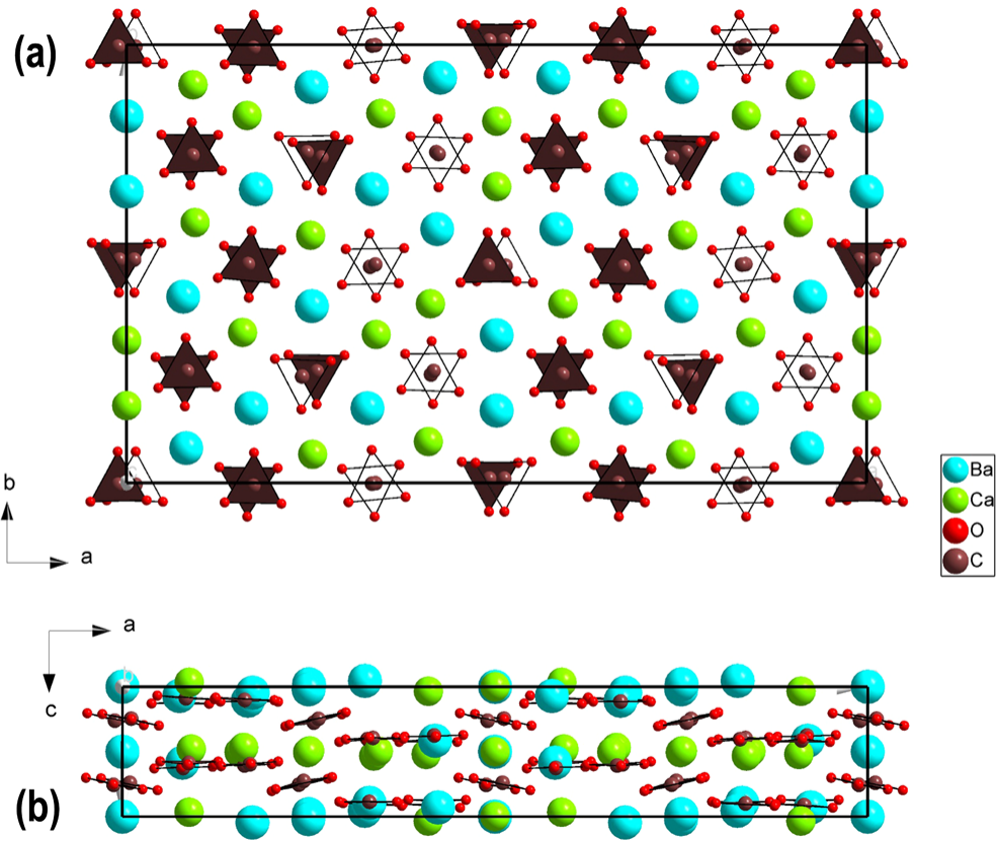
Projections along the *c* and *b* axes of the HP BaCa(CO_3_)_2_*C*2 polymorph found to be stable above 24 GPa. Cyan, green, brown, and small red spheres represent Ba, Ca, C, and O atoms, respectively.

This *C*2 monoclinic unit-cell comes from a translationengleiche subgroup of the initial trigonal *P*321 unit-cell, with the following axes transformation: ***a***′ ∼ 2***a*** + ***b***, ***b***′ ∼ ***b***, ***c***′ ∼ ***c*** and an β angle that could differ from 90°. Calculations predict that this distorted phase would be slightly more stable thermodynamically than the initial alstonite structure above 15 GPa, and suggest that the β angle barely varies around 90° up to 50 GPa. The lengths of the *a*′ and *b*′ axes progressively change their ratio above that pressure but the difference between the length of the pseudotrigonal axes *b*–*a* is relatively small (for instance, *a* and *b* differ in 0.05 Å at 21.3 GPa). According to our calculations, the dominant deformation mechanism under hydrostatic pressure involves slightly correlated tilts of [CO_3_] units located at y/*a*′ ∼ 0, 0.25, 0.5, and 0.75 with respect to the *c*′ axis and small shifts of these units along *a*′. These atomic displacements cause the appearance of 12 different environments around the Ba and Ca atoms in the *C*2 phase (coming from only three environments for each type of atom in the initial *P*321 phase), which evolve independently under compression. This fact produces that the polyhedral volumes slightly diverge above 15 GPa. DFT calculations also predict a second phase transition to a different monoclinic *C*2 phase at 24 GPa. Above this pressure, in addition to the [CO_3_] tilting movements, 1/3 of the carbonate units shift considerably in the *c*′ direction (see Figure 8), which produces a change in the [CO_3_] stacking between cationic layers from a ...2132132... to a ...2112332... configuration (1, 2, and 3 denote here different *z*/*c* values). Upon compression, this atomic rearrangement drives a larger contraction in the *a*′ direction that couples with severe incompressibility in the perpendicular *b*′ direction and causes the increase of the coordination number of Ba atoms from 10 to 11 and 12 (see Figure 7).

The slightly different values for the pseudohexagonal *a* and *b* axes or a very small deviation of the β angle from 90° of the monoclinic cell would produce the apparent widening of the diffraction peaks experimentally observed above 10 GPa. Unfortunately, the huge monoclinic unit-cell generates a large number of reflections that overlap, not allowing the accurate determination of the lattice parameters. The monoclinic lattice parameters that best describe the experimental patterns at different pressures are shown in Table S2. The lattice parameters of the *C*2 phase inferred from our limited quality HP powder XRD data show a splitting of the pseudotrigonal axes above 9 GPa (*b*–*a* ∼ 0.08 Å). Between 9 and 12 GPa, the β angle is approximately 90°, but further compression increases the value to ∼90.6° at *P* > 15 GPa. This monoclinic *C*2 unit-cell was previously proposed by Dickens (in an unpublished study, according to Roberts^55^)^56^ for the ambient conditions alstonite structure. Unfortunately, the crystal structure could only be partially refined and the location of the [CO_3_] groups was uncertain.^56^ However, Dickens′ study provided lattice constants very similar to those of the high-pressure phase found in the present work, i.e., *a* = 30.163(9) Å, *b* = 17.413(5) Å, *c* = 6.110(1) Å, and β = 90.10(1)°, and revealed pseudohexagonal symmetry normal to (001) and alternating Ba- and Ca-rich layers in the ...ABAB... sequence, just like the structure we report here.

A second-order BM-EoS fit to our experimental P–V data above 9 GPa yields a *V*_0_/*Z* = 127.4(6) Å^3^ and a bulk modulus of *B*_0_ = 128(7) GPa. The fact that the structure is considerably less compressible at high pressures is likely due to a combination of two factors: (i) the aforementioned structural behavior, which entails a progressive decrease in compressibility upon compression and (ii) the loss of the hydrostaticity of the pressure-transmitting medium above 10 GPa, which could lead to a change in the slope of the V–P experimental data. Alstonite appears to be extremely sensitive to nonhydrostatic stresses. We also note that our calculations do not reproduce the experimental compressibility above 9 GPa.

## Conclusions

In this work, we have first determined the BaCa(CO_3_)_2_ alstonite structure at ambient conditions from single-crystal XRD measurements. The unit-cell dimensions and atomic coordinates of the heavier metallic atoms coincide with those recently reported by Bindi et al.,^20^ but the location of some of the carbonate groups is different. This entails different coordination environments in the Ba and Ca atoms despite being 10-fold and 8-fold coordinated, respectively, in both structural models. The atomic weight of the cations present and the crystal structure give a density value for the alstonite mineral at ambient conditions of 3.668 g/cm^3^, following the density against cation atomic weight trend observed in carbonates where the coordination number of cations by oxygen atoms is higher than 6.^57^

It is known that the ordered combination of two types of cations in a carbonate, like the one which occurs in alstonite, makes a more stable carbonate than the single-cation end-members. This becomes clear from direct comparison of the free energies of formation from ions of CaCO_3_ calcite/aragonite, BaCO_3_ witherite, and BaCa(CO_3_)_2_ alstonite.^57^ The relative stability of the different BaCa(CO_3_)_2_ polymorphs, however, was never investigated. Our DFT calculations show that barytocalcite is the thermodynamically stable phase but the differences in enthalpies with alstonite and paralstonite are very small (<0.08 eV/formula unit), which suggests that either of these phases could be found in nature, as in fact occurs. The alstonite structure previously reported^20^ appears to be less stable at ambient conditions.

Subsequently, we have experimentally and theoretically studied the phase behavior of BaCa(CO_3_)_2_ alstonite at high pressure. Its compressibility is strongly anisotropic. The highly incompressible [CO_3_] carbonate groups are arranged perpendicular to the *c* axis, which makes this axis approximately 3 times more compressible than the *a* axis. The bulk modulus of alstonite (*B*_0_ = 60(3) GPa) is approximately an average of the bulk modulus for their corresponding single-cation minerals. Above 9 GPa, experiments reveal that the trigonal *P*321 phase of alstonite transforms into a monoclinic phase, with no volume discontinuity at the transition. The structure suffers a distortion, which can be described by the *C*2 space subgroup of the initial phase and two slightly different pseudotrigonal *a* and *b* axes. This is consistent with DFT results, which additionally predict that, upon further compression, at 24 GPa, the carbonate groups within the structure rearrange and the coordination number of the Ba atoms increase to 11–12. This second high-pressure monoclinic (also *C*2) polymorph is an alstonite structural variant more stable than barytocalcite at high pressures.

Carbonates play a central role in the subduction transport of oxidized carbon from the earth’s surface to the mantle. Although at upper mantle conditions the chemistry of carbonates within subducting slabs is thought to be mainly restricted to the CaCO_3_–MgCO_3_–FeCO_3_ system, the study of the effect of the inclusion of larger cations in the carbonate structure is important. Our results provide information on the structural local environment of metal atoms in a double Ca–Ba carbonate, which affects the density and the solubility, and reports the phase stability and compressibility of this carbonate upon compression. More thorough structural analyses of carbonate minerals will give further insight into potential chemical substitution at inner earth conditions and the great mineralogical diversity found in nature.

## Supporting Information Available

Tables showing the experimental and calculated (static) lattice parameters and unit-cell volumes for BaCa(CO_3_)_2_ alstonite at different pressures (PDF)
