# Supplementary material for: Crystal Structure of BaCa(CO3)2 Alstonite Carbonate and Its Phase Stability upon Compression
Source: ACS Earth Space Chem. 2021 Apr 23;5(5):1130–9. doi: 10.1021/acsearthspacechem.1c00032 (PMC8656406; doi:10.1021/acsearthspacechem.1c00032)
Supplement: Supplementary file 1 — sp1c00032_si_001.pdf [file sp1c00032_si_001.pdf]

Supplementary Material

of

Crystal structure of $BaCa(CO_3)_2$ alstonite carbonate and its phase stability upon compression

Raquel Chuliá-Jordán[1,*], David Santamaria-Perez[1], Javier Ruiz-Fuertes[2], Alberto Otero-de-la-Roza[3], Catalin Popescu[4]

[1] Departamento de Física Aplicada-ICMUV, Universitat de València, MALTA Consolider Team, 46100 Valencia, Spain

[2] DCITIMAC, Universidad de Cantabria, MALTA Consolider Team, 39005 Santander, Spain

[3] Departamento de Química Física y Analítica, Facultad de Química, Universidad de Oviedo, MALTA Consolider Team, 33006, Oviedo, Spain

[4] CELLS-ALBA Synchrotron Light Facility, Cerdanyola del Vallès 08290, Barcelona, Spain

Tables collecting the experimental and calculated (static) lattice parameters and unit cell volumes for $BaCa(CO_3)_2$ alstonite at different pressures.

Tabla S1.- Lattice parameters (*a* and *c*) and unit cell volume of *P*321 $BaCa(CO_3)_2$ alstonite at different pressures. Pressure uncertainties are estimated to be ~0.05 GPa below 10 GPa, and ~0.1 GPa above this pressure.

Pressure (GPa)	a (Å)	c (Å)	V (Å^3)
10^{-4} (single crystal)	17.449 (3)	6.125 (2)	1615.0 (7)
1.25	17.336 (4)	6.046 (3)	1573.6 (9)
1.30	17.337 (4)	6.047 (3)	1574.1 (9)
1.40	17.333 (4)	6.039 (3)	1571.1 (9)
1.45	17.339 (4)	6.049(3)	1574.9 (9)
1.55	17.330 (4)	6.043 (3)	1571.8 (9)
1.70	17.318 (4)	6.024 (3)	1564.5 (9)
1.85	17.319 (4)	6.027 (3)	1565.6 (9)
2.00	17.297 (4)	6.011 (3)	1557.5 (9)
2.15	17.303 (4)	6.010 (3)	1558.2 (9)
2.50	17.274 (4)	5.985 (3)	1546.5 (9)
2.65	17.277 (4)	5.981 (3)	1546.2 (9)
3.05	17.244 (4)	5.956 (3)	1533.7 (9)
3.25	17.249 (4)	5.954 (3)	1534.1 (9)
3.50	17.225 (4)	5.939 (3)	1526.1 (9)
3.65	17.226 (4)	5.934 (3)	1525.0(9)
4.20	17.192 (4)	5.904 (3)	1511.2 (9)
4.30	17.192 (4)	5.900 (3)	1510.2 (9)
4.55	17.176 (4)	5.892 (3)	1505.2 (9)
4.70	17.174 (4)	5.886 (3)	1503.4 (9)
5.35	17.144 (4)	5.852 (3)	1489.5 (9)
5.65	17.142 (4)	5.855 (3)	1489.9 (9)
6.10	17.111 (4)	5.822 (2)	1476.2 (9)
6.45	17.114 (4)	5.817 (2)	1475.4 (9)
7.00	17.077 (4)	5.783 (2)	1460.5 (8)
7.30	17.080 (4)	5.782 (2)	1460.8 (8)
8.05	17.038 (4)	5.744 (2)	1443.9 (8)
8.40	17.037 (4)	5.746 (2)	1444.5 (8)
8.80	17.021 (4)	5.722 (2)	1435.4 (8)
9.70	17.013 (4)	5.696 (2)	1427.8 (8)
10.3	17.007 (4)	5.675 (2)	1421.5 (8)
11.1	17.004 (4)	5.657 (2)	1416.4 (9)
12.0	16.978 (4)	5.638 (2)	1407.4 (8)
12.5	16.990 (4)	5.616 (4)	1403.9 (8)
13.6	16.972 (4)	5.596 (4)	1395.8 (9)
14.1	16.987 (5)	5.576 (4)	1393.3 (10)
14.7	16.990 (5)	5.566 (4)	1391.4 (10)
15.3	17.003 (5)	5.564 (4)	1393.2 (10)
15.6	16.993 (5)	5.531 (4)	1383.2 (10)
16.0	16.990 (5)	5.508 (4)	1376.9 (10)
16.7	16.989 (5)	5.503 (4)	1375.6 (10)
17.5	16.995 (5)	5.462 (4)	1366.3 (10)
17.6	16.982 (5)	5.457 (4)	1362.9 (10)
18.1	16.986 (5)	5.441 (4)	1359.7 (10)
0.05 (recovered)	17.408 (4)	6.115 (3)	1604.7 (9)

Tabla S2.- Lattice parameters (*a*, *b*, *c* and β angle) and unit cell volume of *C*2 $BaCa(CO_3)_2$ alstonite at different pressures above 9 GPa. The a_{trig} is the pseudo-trigonal lattice parameter calculated as $\vec{a} = \frac{\vec{a'} - \vec{b'}}{2}$. Pressure uncertainties are estimated to be ~0.05 GPa below 10 GPa, and ~0.1 GPa above this pressure.

Pressure (GPa)	*a'* (Å)	*b'* (Å)	*c'* (Å)	β angle (º)	V (Å³)	a_{trig} (Å)
8.40	29.496(5)	17.029(3)	5.746(2)	90.01(3)	2886(2)	17.030(3)
8.80	29.425(5)	17.066(3)	5.711(2)	90.00(3)	2868(2)	16.989(3)
9.70	29.373(5)	17.060(3)	5.690(2)	90.03(3)	2851(2)	16.959(3)
10.3	29.370(5)	17.055(3)	5.672(2)	90.00(3)	2841(2)	16.957(3)
11.1	29.404(6)	17.047(3)	5.656(3)	90.00(3)	2835(2)	16.976(3)
12.0	29.360(6)	17.049(3)	5.646(3)	90.00(3)	2826(2)	16.951(3)
12.5	29.407(6)	17.022(3)	5.621(3)	90.00(3)	2813(2)	16.978(3)
13.6	29.377(6)	17.039(3)	5.604(3)	90.27(5)	2805(2)	16.961(3)
14.1	29.352(6)	17.027(3)	5.586(3)	90.46(8)	2792(2)	16.946(3)
14.7	29.359(7)	17.030(4)	5.572(3)	90.57(7)	2785(3)	16.950(4)
15.3	29.359(7)	17.032(4)	5.557(3)	90.61(7)	2779(3)	16.950(4)
15.6	29.339(7)	17.042(4)	5.543(3)	90.65(7)	2772(3)	16.939(4)
16.0	29.332(7)	17.009(4)	5.533(3)	90.78(10)	2766(3)	16.935(4)
16.7	29.308(7)	17.044(4)	5.505(3)	90.78(8)	2749(3)	16.921(4)
17.5	29.326(7)	17.046(4)	5.478(3)	90.75(10)	2738(3)	16.931(4)
17.6	29.228(7)	17.064(4)	5.478(3)	90.63(9)	2732(3)	16.875(4)
18.1	29.183(7)	17.049(4)	5.464(3)	90.59(10)	2719(3)	16.849(4)
